# Notes and News

**Published:** 1902-07-05

**Authors:** 


### Notes and News.

The Harveian Lectures, in November, will be given by Mr. Alban Doran, on Uterine Fibroids, considered from a clinical and surgical standpoint.

Hard as is the struggle for existence in the Old World it seems to be even worse in the New, if we may judge by the number of those who retire from the fight by way of suicide; Chicago, which claims to be the most progressive of the cities of this world, also standing at the head of the list in regard to the prevalence of suicide.

The Board of Trade report on the sight tests used in the mercantile marine states that of the 24 candidates who failed in form vision during 1901 one was re-examined; while of the 50 who failed in colour vision 14 were re-examined on appeal, and of these four passed and ten were rejected. Of the candidates who had failed during the previous year, 1900, three were re-examined in colour vision; all three again failed. The number of officers already in possession of certificates who, on coming up for examination in the year 1901, failed to pass the sight tests, was one. This was a first mate, who failed in form vision; but he subsequently passed on re-examination.

Although there has been no Coronation the honours list is a long one. Among those mentioned we note that Lord Lister, F.R.S., has been made a Privy Councillor; Sir Francis Laking, K.C.V.O., M.D., and Sir Frederick Treves, K.C.V.O., C.B., F.R.C.S., have had baronetcies conferred upon them; that the honour of knighthood has been conferred upon Dr. W. J. Collins, Mr. Alfred Cooper, Dr. J. Halliday Croom, Dr. Conan Doyle, Dr. Thomas Fraser, F.R.S., Mr. Victor Horsley, F.R.S., Mr. H. G. Howse, Mr. H. B. Longhurst, Professor William Macewen, F.R.S., Dr. Isambard Owen, and Professor Rücker, F.R.S. Sir William Church, Bart., and Professor William Ramsay, F.R.S., have been made Knights Commanders, and Major Ronald Ross, F.R.S., M.D., and Dr. Whitelegge, Companions of the Order of the Bath. Lord Lister has also been appointed to the new "Order of Merit" which has been instituted by the King.

Princess Louise (Duchess of Argyll) will open the London County Council's Farmfield Home for Inebriates some day in October next.

It is stated that Major Ronald Ross, F.R.S. has decided not to take up the duties of the office at the Jenner Institute of Preventive Medicine to which he was recently appointed.

The Society for the Study of Disease in Children held their provincial meeting at Manchester on Saturday, June 21. Cases were demonstrated in the wards of the Children's Hospital, Pendlebury, by the honorary medical staff prior to the meeting, which was well attended. Dr. Henry Ashby was in the chair, and many London representatives and provincial members were present. The provincial meeting next year will take place at Brighton.

The Duke and Duchess of Aosta, attended by Colonel Recli, the Marquis and Marchioness Torrigiani, Captain Durini, and General G. Ramsay Slade, C.B., visited the Italian Hospital, Queen Square, last week. Their Royal Highnesses were received by the Italian Ambassador (president of the hospital), accompanied by Mme. Pansa, and, the members of the committee of management and of the medical staff having been presented to the Duke and Duchess, a tour of the building took place.

The *Boston Medical and Surgical Journal* publishes some curious statistics of a case in which criminal tendencies appeared to be transmitted to a large number of the descendants of particular individual in whom these tendencies were well marked. The woman in question was addicted to vicious habits, she kept a disreputable house, she drank to excess, and died at the age of 51 in the year 1827. Her descendants number 800, and 700 of them have been convicted as criminals, some having been convicted more than once. There have been among them 342 drunkards and 127 immoral women, and, astonishing to relate, in this wretched family there have been 37 murderers who have been executed for their crimes.

A curious case of what we suppose some people would call moral insanity came before the Central Criminal Court last Monday. A nurse girl, aged 16, was indicted for attempting setting fire to a dwelling house. At first she denied it, but ultimately admitted it and pleaded guilty. It appeared that she had put some of the children alone in an upper room and then set fire to a bed in another room. The flames were extinguished, but not before damage amounting to £25 had been done. In passing sentence Mr. Justice Darling said that it might be only her misfortune, but when she was in a former situation the house caught fire, and in another place a child was burnt to death, while she was discharged from a subsequent situation for cruelty to a child. He thought that it must be a malady of some kind that she was suffering from, and he sentenced her to three years' penal servitude, remarking that she would be taken care of.

